# Praziquantel–Clays as Accelerated Release Systems to Enhance the Low Solubility of the Drug

**DOI:** 10.3390/pharmaceutics12100914

**Published:** 2020-09-24

**Authors:** Ana Borrego-Sánchez, Rita Sánchez-Espejo, Fátima García-Villén, César Viseras, C. Ignacio Sainz-Díaz

**Affiliations:** 1 Instituto Andaluz de Ciencias de la Tierra, CSIC-University of Granada, Av. de las Palmeras 4, 18100 Granada, Spain; ritase@correo.ugr.es (R.S.-E.); ignacio.sainz@iact.ugr-csic.es (C.I.S.-D.); 2 Department of Pharmacy and Pharmaceutical Technology, Faculty of Pharmacy, University of Granada, Campus de Cartuja s/n, 18071 Granada, Spain; fgarvillen@ugr.es (F.G.-V.); cviseras@ugr.es (C.V.)

**Keywords:** praziquantel, drug, montmorillonite, sepiolite, organic solvents, in vitro dissolution tests, cytotoxicity

## Abstract

Praziquantel is an antiparasitic drug indicated for the treatment of the schistosomiasis disease. This drug has very low aqueous solubility, requiring high oral doses for its administration which gives rise to side effects, therapeutic noncompliance and the appearance of resistant forms of the parasite. Clay minerals, like sepiolite and montmorillonite, are innocuous, non-toxic, biocompatible and low-cost excipients. Additionally, clays have high adsorbent properties that allow them to encapsulate drugs in nanometric spaces present in the channels in the case of the sepiolite or between the layers in the case of the montmorillonite. The interactions between the drug and clay minerals are studied experimentally with the strategy for preparing interactions products in organic solvents (ethanol, acetonitrile and dichloromethane) so that the interaction will be more effective and will be enhanced the aqueous solubility of praziquantel. The results showed that in the interaction products, the drug interacted with both clay minerals, which produced the loss of the crystallinity of the drug demonstrated by different techniques. This led to a significant increase in the dissolution rate of the praziquantel in all the interaction products in the simulated gastrointestinal tract media, except for the praziquantel–montmorillonite product prepared in dichloromethane that presented a controlled release in acid medium. Moreover, in vitro cytotoxicity and cell cycle studies were performed in the interaction products prepared with ethanol. The interaction product with sepiolite was biocompatible with the HTC116 line cells, and it did not produce alterations in the cell cycle. However, interaction products with montmorillonite did not produce cell death, but they showed affectation and damage of cells in the cell cycle study at the highest concentration tested (20–100 µM). Therefore, the different organic solvents used are adequate for the improvement of the biopharmaceutical profile of praziquantel. Drug–clay interaction products, specifically with sepiolite, showed very promising results in which new accelerated oral release systems of the praziquantel were obtained.

## 1. Introduction

Neglected tropical diseases are infectious diseases that affect a sixth of the world population, more than a billion people, mainly in poor populations of tropical countries in Africa, Latin America, Asia and the Middle East. Their prevalence increases with the absence of salubrious water and adequate sanitation conditions and the most vulnerable population are children in contact with contaminated water [1].

Schistosomiasis is the second neglected tropical disease with the highest prevalence, widespread mainly in tropical Africa. Therefore, the improvement of the existing treatment with the praziquantel (PZQ) drug is very important for saving lives. Since schistosomiasis is in the expansion phase and currently affects 240 million people in the world, of which only 88 million receive adequate treatment, it is estimated that it produces more than 200,000 deaths annually, and it is extended in 78 countries around the world, mainly in the tropical and subtropical region, being in Africa the second most prevalent disease in children. This disease is acquired by contact with fresh water infested with larvae of the parasite [2,3,4,5].

PZQ is the recommended treatment against all forms of schistosomiasis. It has high efficacy, low toxicity and is administered orally. In addition, the cost of production and sale of PZQ is low [2,6]. However, it has a few disadvantages. First, it is a racemic compound, with only one of its enantiomers being pharmacologically active [7,8]. In addition, it is a Class II drug of the Biopharmaceutical Classification System (BCS) and has very low aqueous solubility and high permeability, so that the dissolution is the factor limiting the rate of absorption [9,10,11]. Its low solubility in water makes it necessary to administer high oral doses, which gives rise to secondary effects that increase the therapeutic noncompliance and favor the appearance of resistant forms of the parasite. For these reasons, the use of low-cost natural inorganic excipients was proposed as a starting hypothesis to improve the aqueous solution profile of the PZQ, maintaining the final cost of the drug, which is very important given the population to which it is subject destined. 

Montmorillonite and sepiolite are innocuous, non-toxic, biocompatible and low-cost clay minerals. Additionally, clays have high adsorbent properties that allow them to encapsulate drugs in nanometric spaces present between the layers in the case of the montmorillonite or in the channels in the case of the sepiolite [12,13,14,15,16,17]. 

The interaction between PZQ and other excipients was also studied in order to improve the low solubility of the drug, for example, with calcium carbonate [18,19,20], β-cyclodextrins [21,22,23], polyvinyl-pyrrolidone [24,25], polyethylene glycols [26,27,28], sodium starch glycolate [29], phosphatidylcholine–containing liposomes [30], layered double hydroxides [31], among others. Specifically, the interaction between PZQ and montmorillonite in aqueous medium was previously studied, although no improvements of in vitro dissolution and absorption rates were observed [32]. The lack of relevant biopharmaceutical improvements was explained by the absence of PZQ in the interlayer space of montmorillonite. Water molecules present in the clay interlayer space would be blocking the entrance of the drug, thus PZQ would be solely absorbed in the external surface of montmorillonite. Therefore, in our previous studies [33,34], the interactions between PZQ and montmorillonite and sepiolite in absence of water were studied as a strategy for enhancing the aqueous solubility of the drug. To do this, ethanol 99% (*v*/*v*) was used as a solvent increasing the interaction between the drug and the excipient. The results showed a great increase in the dissolution rate of the drug and the total amount of drug dissolved in vitro in the interaction products, due to the high interaction between both components. Therefore, this new procedure was found to be an effective strategy to improve the biopharmaceutical profile of the PZQ. 

Hence, in this work, the preparation of interaction products is studied following the previously described procedure [33,34] comparing the use of different organic solvents, like ethanol, acetonitrile and dichloromethane. The aim is to prepare and characterize these systems and, by these means, to determine the potential influence of the organic solvent on the release and solubility of the drug. In particular, the results were compared with those obtained with ethanol in which a significant improvement in the biopharmaceutical profile was described. As well as this, in vitro cytotoxicity and cell cycle studies are carried out for checking if these systems are biocompatible and suitable for human use.

## 2. Materials and Methods 

### 2.1. Materials

Pristine racemic PZQ drug was purchased from Sigma Aldrich (St. Louis, MO, USA). Purified pharmaceutical degree. Montmorillonite (Veegum HS^®^, VHS, Norwalk, CT, USA) was purchased from Vanderbilt Company (Norwalk, CT, USA). The Sepiolite samples from Vicálvaro (Madrid) (SEP) were kindly gifted by TOLSA (Madrid, Spain). Ethanol of 99% (*v*/*v*) of purity, acetonitrile, and dichloromethane were used as solvents.

### 2.2. Methods

#### 2.2.1. Preparation of Praziquantel–Clay Minerals Interaction Products

PZQ was put in contact with montmorillonite and sepiolite clay powders in 100 mL of different solvents: ethanol 99% (*v*/*v*), acetonitrile, and dichloromethane, in such way that the clay/drug ratio was 5:1 *w*/*w*. The dispersion was put into magnetic stirring for 24 h at room temperature. After 24 h, the solvent was evaporated at 40 °C using a rotary evaporator (Buchi^®^ R II, Flawil, Switzerland). The solid residue was stored in a desiccator at room temperature with freshly activated silica-gel grafted with a moisture indicator of Co salt. 

The interaction products obtained were: PZQ–VHSet prepared with VHS and dispersed in ethanol, PZQ–VHSac prepared with VHS and dispersed in acetonitrile, PZQ–VHSdic prepared with VHS and dispersed in dichloromethane, PZQ–SEPet prepared with SEP and dispersed in ethanol, PZQ–SEPac prepared with SEP and dispersed in acetonitrile and PZQ–SEPdic prepared with SEP and dispersed in dichloromethane.

#### 2.2.2. X-ray Diffraction

Powder X-ray diffraction was performed with equipment of Panalytical X’Pert Pro (Marvel Panalytical, Madrid, Spain) model, which is managed by a fully computerized system. It also has an automatic charger system and uses the X’Celerator detector (Marvel Panalytical) (linear type, detects the RX along a line) with the Cu Kα wavelength that allows a quick and accurate study. The information of diffraction was analyzed using X’Pert HighScore^®^ software (Version 2.2., Marvel Panalytical, Madrid, Spain).

#### 2.2.3. Thermal Analysis

Differential Scanning Calorimetric analysis (DSC) and Thermogravimetric Analysis (TGA) were performed with a Mettler Toledo mod. TGA/DSC1 calorimeter (Mettler Toledo, Barcelona, Spain). The TGA equipment sensor is a thermostatic microbalance with an accuracy of 0.1 μg, which is the limit established for weighing precision. On the other hand, the TGA/DSC1 has sensors that obtain the DSC signal directly and in real-time.

The TGA was performed in air using a heating rate of 10 °C/min in the 30–400 °C temperature range and the DSC runs were performed with a heating rate of 2 °C/min and nitrogen was used as a purge gas in DSC under the flow of 15 mL/min.

#### 2.2.4. Fourier Transform Infrared (FTIR) Spectroscopy

FTIR spectra were performed with the JASCO 6200 spectrophotometer (JASCO, Pfungstadt, Germany) which has an attenuated total reflectance (ATR) accessory. Measurements were performed in the range 4000–600 cm^−1^ with a 0.5 cm^−1^ resolution and a well-plate sampler. The results were analyzed using the Spectra Manager II software (Version 2, JASCO). 

#### 2.2.5. In Vitro Release Studies

In vitro dissolution tests were performed according to the European Pharmacopoeia procedures dealing with solid oral dosage forms. In particular, the paddle apparatus (apparatus 2, Sotax AT7, Teknokroma^®^, Barcelona, Spain) with sinkers was set at 150 rpm and 37 °C, filled with 1 L of dissolution medium and working at sink conditions. Stomach physiological fluid was simulated by using a dissolution medium composed of HCl 0.001 M, while simulated intestinal fluid (SIF) was used with a phosphate buffer (pH = 6.8) without enzymes.

Hard gelatin capsules were subjected to the in vitro dissolution test. The capsules were prepared with 210 mg of each PZQ–VHS and PZQ–SEP interaction product, in which 35 mg corresponds to PZQ. Each of these interaction products (PZQ–VHS and PZQ–SEP) were prepared with the different solvents previously explained. Similarly, hard gelatin capsules containing 35 mg of PZQ were elaborated as reference. 

During the in vitro dissolution tests, at predetermined time intervals, 5 mL of samples were withdrawn, the volume immediately replenished with fresh dissolution medium. The drug amount was quantified by High-Performance Liquid Chromatography (HPLC) after being filtered through 0.45 μm nitrocellulose membranes (Merck-Millipore^®^, Darmstadt, Germany). HPLC was performed in a 1260 Infinity II HPLC (Agilent Technologies Inc., Santa Clara, CA, USA) equipped with a quaternary pump, autosampler, column oven and UV-VIS diode-array spectrophotometer. The stationary phase was a Lichrospher^®^ (100 RP-18, C18, 5 μm, 15 cm × 3.2 mm) column (Merck Millipore^®^, Darmstadt, Germany). Isocratic conditions were used, the mobile phase formed by a mixture of H_2_O and CH_3_CN (35:65 *v*/*v*). The flow rate was set at 0.8 mL/min with a sample injection volume of 10 μL. PZQ was detected at 225 nm during the 5 min of the run time of the HPLC analysis. The software LC Open LAB HPLC 1260 (Agilent Technologies Inc.) was used to record and treat the chromatograms. The linear analytical method was obtained in the concentration range of 5 to 100 mg/L of PZQ in both dissolution media (correlation coefficients of 1 in both HCl 0.001 M and in SIF). 

Subsequently, release data fitting was performed using kinetic models intended to describe the drug release from immediate or modified dosage forms [35,36,37]. These models, such as zero order, first order, cube root, Higuchi, Peppas, and Weibull, were used to fit experimental data obtained from in vitro dissolution studies. The fitting was carried out linearizing the equations. The choice of the best model to describe the dissolution/release process of the drug was based on the correlation coefficient (R^2^) criterium. A second evaluation criterium that is frequently used is called the Akaike Information Criterion (AIC) [38,39]. Herein, it is also considered. 

#### 2.2.6. Solubility Studies

The solubility of PZQ, PZQ–VHSet and PZQ–SEPet, which was used as a reference for the rest of the interaction products, were obtained in both HCl 0.001 M and SIF (phosphate buffer at pH = 6.8, without enzymes). PZQ solubility was obtained by placing 30 mg of drug in 10 mL of HCl and SIF (supersaturated conditions). The solution was stirred at 37 °C for 24 h (thermostatic bath). Then, the solution was centrifuged, the supernatant filtered (nitrocellulose membranes, 0.45 μm, Merck Millipore^®^) and quantified by HPLC (Agilent Techonologies Inc.). The amount of dissolved drug, determined by the chromatography, corresponds to PZQ solubility in the corresponding medium. In order to demonstrate the solubility improvement of PZQ in the composites (PZQ–VHSet and PZQ–SEPet), drug solubility was determined with the very same procedure. Triplicate experiments were performed in all samples. 

#### 2.2.7. Cell Culture

Cellular viability and cell cycle profiles were studied over a human colorectal carcinoma cell line HCT 116 (ATCC^®^ CCL-247™, Manassas, VA, USA). HCT 116 cells were cultured in Dulbecco’s Modified Eagle’s Medium (DMEM, Gibco™, Dublin, Ireland) supplemented with 10% *w*/*w* of Fetal Bovine Serum (FBS, Gibco™, Dublin, Ireland), 1% of Glutamax (BioWhittaker^®^, Cologne, Germany) and 1% of penicillin/streptomycin (BioWhittaker^®^). Cells were cultured in an incubator set at 37 °C and 5% of CO_2_.

#### 2.2.8. Cytotoxicity Studies

Cytotoxicity studies of PZQ, VHS and SEP were performed by using PZQ veterinary antiparasitic doses. Consequently, the amount of mineral (VHS and SEP) that should be subjected to study was five times higher than that of PZQ. To do so, intermediate dilutions of 100 mM and 10 mM were prepared in dimethyl sulfoxide (DMSO). The same procedure and concentrations were used for cytotoxicity studies of PZQ–VHSet and PZQ–SEPet. 

AlamarBlue™ bioassay (Thermo Fisher Scientific, Carlsbad, CA, USA) was used to study cell proliferation. To do so, HCT 116 cells were seeded in 96-well plates (density of 3–6 × 10^4^ cells/cm^2^) in a final DMEM volume of 200 μL. The corresponding solid samples were subsequently added (PZQ, VHS, SEP, IP PZQ–VHSet, or IP PZQ–SEPet). All samples were tested in a wide range of concentrations (100 μM, 20 μM, 4 μM, 800 nM, 160 nM, 32 nM, and 6.4 nM) and three replicates were used for each experience. Then, the well plates were incubated at 37 °C, 5% CO_2_ for 48 h. At the end of contact time, 10 μL of cell viability reagent (PrestoBlue™, Thermo Fisher Scientific) was added to each well. The PrestoBlue™ reagent is a resazurin compound that works as a cellular viability indicator since its color changes from blue to pink when reduced by living cells. After 15 min of incubation in presence of PrestoBlue™ reagent, fluorescence was quantified at 535–90 nm in a microplate reader (Tecan, Zürich, Switzerland). 

#### 2.2.9. Cell Cycle Studies

HCT 116 cells were cultured in 24 well-plates (250,000 cells/well) in a final DMEM volume of 500 μL. In these conditions, cells were subjected to PZQ, VHS, SEP, IP PZQ–VHSet, and IP PZQ–SEPet samples for 48 h. Increasing concentrations of each sample (0.8 μM, 4 μM, 20 μM, and 100 μM) were used. Cells entering the apoptotic or necrotic cycle were detected with a propidium iodide staining procedure, as explained in a previous protocol [40]. Briefly, once the cells were in contact with each sample, they were collected and washed with 2 mL of phosphate-buffered saline (PBS) at 4 °C and subsequently fixed with ethanol 70° (V_f_ = 1 mL, diluted 1:9 in PBS) for 5 min, while maintained on ice. Ethanol was withdrawn and fixed cells were washed with PBS. Then, they were resuspended in a solution consisting of 250 μL of PBS and 250 μL of a DNA extraction solution (0.2 M Na_2_HPO_4_, 0.1 M C_6_H_8_O_7_, pH 7.8) for 10 min at 37 °C. Then, the supernatant removed and 200 μL of the staining solution were added (8 μL propidium iodide (1 mg/mL) and 2 μL RNAse 100 (μg/mL)), the cells further incubated in the dark at 37 °C for 10 min. A FACScalibur cytometer (Becton Dickinson and Co., Franklin Lakes, NJ, USA) was used to measure the fluorescence (FL2 detector). Sub-G1 cells (dead cells, that is, cells that entered either necrotic or apoptotic phases) were detected by the Cell Quest software (Version 1.0.2, BD, Biosciences, Franklin Lakes, NJ, USA).

## 3. Results

### 3.1. X-ray Diffraction

**Figure 1:**
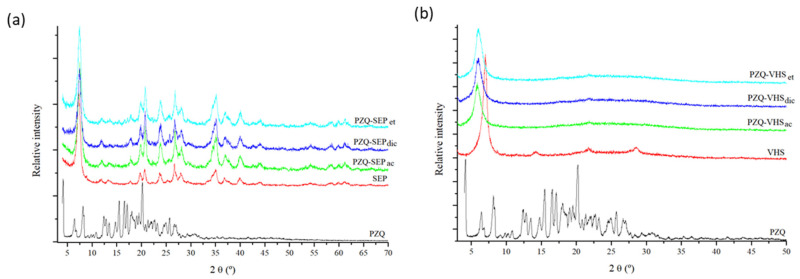
XRPD patterns of the studied samples with sepiolite (**a**) and montmorillonite (**b**).

The interaction products were prepared following the methodology described above in drug–clay. These materials and raw materials were characterized by powder XRD, showing the results in Figure 1. PZQ drug revealed the most intense reflection at around 16.5° (2θ units) and other intense reflections at approximately 8.0°, 19.1° and 23.3° (2θ units) in accordance with that previously reported [41].

After interaction with the clay minerals, a practically complete absence of drug reflection was observed, probably due to a loss of crystallinity of PZQ (Figure 1). In previous works, similar behavior of PZQ was observed in the formation of a solid dispersion with polyvinylpyrrolidone [24], with sodium starch glycolate [29], and with glycyrrhizic acid–forming micelles [42].

The interaction product of PZQ with sepiolite using different solvents in the preparation method (Figure 1a) showed an XRD pattern similar in all the cases. In the profiles, clear peaks of the PZQ drug were not observed; only the peaks of sepiolite were observed. Therefore, after the interaction, a loss of drug crystallinity was found. 

In the oriented aggregate powder X–ray diffraction pattern of the interaction products of PZQ with montmorillonite (Figure 1b), the peaks of PZQ were not observed either in any of the products. However, the (001) reflection peak of the VHS increased in the interaction products, confirming the intercalation and the presence of PZQ in the interlayer space of the clay. 

The interaction product PZQ–VHSet showed an increase in the interlayer space of montmorillonite from 1.26 nm to *d*(001) = 1.50–1.61 nm in accordance with that reported previously [33,43]. The interaction product PZQ–VHSdic increased to *d*(001) = 1.47 nm; and the spacing of PZQ–VHSac increased to *d*(001) = 1.51 nm (Figure 1b). These results corroborated that the PZQ is present in the interaction products and the peaks of PZQ were not observed probably due to the complete intercalation of the drug in the interlayer space of the clay and the loss of crystallinity of the drug. The PZQ molecules intercalate in the confined space of montmorillonite as individual molecules where no recrystallization is possible. Nevertheless, the *d*(001) reflection peaks are wider in the interaction products than in the pristine VHS. This indicates than a range of different *d*(001) spacings can be produced, especially in PZQ–VHSdic, where two peaks can be distinguished. In previous modeling studies, we observed the formation of a monolayer of PZQ in the interlayer space of VHS, where different densities of the drug can be found and several conformations of the adsorbate molecule can happen [44]. These differences can be responsible for the variation of *d*(001) spacing widening this (001) peak, maintaining the monolayer configuration.

### 3.2. Thermal Analysis

**Figure 2:**
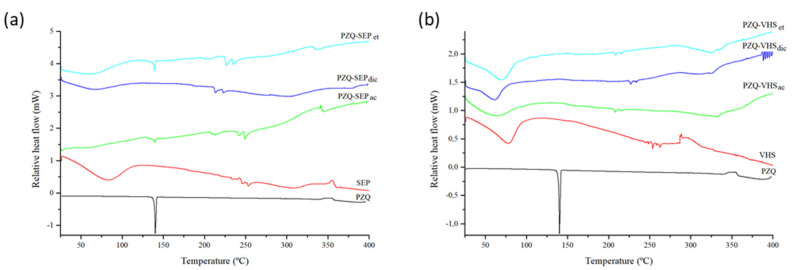
Differential Scanning Calorimetric (DSC) profiles of sepiolite and interaction products of sepiolite (**a**) and montmorillonite and interactions products of montmorillonite (**b**).

The DSC profiles of the pristine PZQ showed a strong endothermic peak at 144 °C, according to previous work [18] (Figure 2). The DSC profile of SEP and VHS indicated broad endothermic peaks in the range 50–90 °C due to the evaporation of water (Figure 2).

In Figure 2a, the PZQ–SEPet and PZQ–SEPac interaction product profiles presented a very low-intensity peak corresponding to the melting of the drug. This indicates that a small amount of PZQ crystallized outside of the channels in the interstitial spaces of solid. Specifically, the enthalpy of the endothermic peak (ΔH) at 144 °C was 3.9 and 1.3 J g^−1^ respectively, which can be compared with the enthalpy of 90.4 J g^−1^ of the melting peak of the pristine drug. Therefore, the ΔH melting (PZQ–SEP)/ΔH melting (pure PZQ), was 4.3% in PZQ–SEPet and 1.4% in PZQ–SEPac. On the contrary, in the PZQ–SEPdic profile, the complete disappearance of the melting peak of the drug was observed. 

The DSC curves of PZQ–VHS interaction products showed the water evaporation of the raw materials until 100 °C, and no melting peak of PZQ was observed in all interaction products (PZQ–VHSet, PZQ–VHSdic and PZQ–VHSac) (Figure 2b). 

Therefore, the lack of the peak of PZQ in the interaction products was observed probably due to the amorphization and no recrystallization of the drug after the complete interaction with the clay. This justified the lack of drug reflections in the PZQ–clay powder XRD patterns. Previous studies found similar results [21,31,33].

In summary, the appearance of a small peak of the drug in some of the samples is practically insignificant and its appearance or non-appearance depends on the variability of production, compared with previous results [33]. Therefore, in drug–clay interaction products, a practically complete amorphization of the drug occurs.

**Figure 3:**
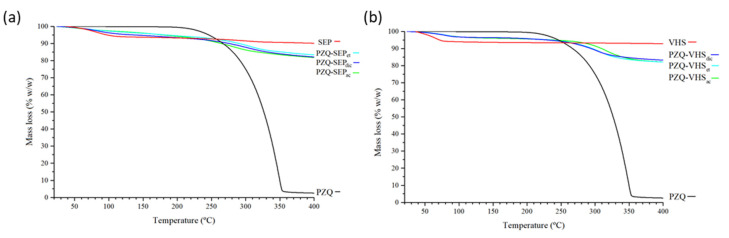
Thermogravimetric Analysis (TGA) profiles of the studied samples with sepiolite (**a**) and with montmorillonite (**b**).

According to the first weight loss of the TGA profile, absorbed water of VHS and SEP accounted for 8% and 10% *w*/*w*, respectively (Figure 3). Zeolitic water loss of SEP occurred at 250–350 °C [45]. 

In the interaction products both with sepiolite and montmorillonite, thermal decomposition of the drug resulted in weight losses in the range between 200 and 400 °C, corroborating the adsorption of the PZQ in these mineral solids (Figure 3).

### 3.3. Fourier Transform Infrared Spectroscopy

**Figure 4:**
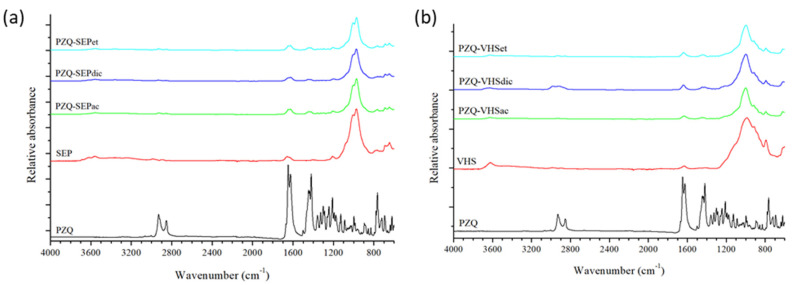
Fourier Transform Infrared (FTIR) spectra of sepiolite products (**a**) and of montmorillonite products (**b**).

FTIR spectra of PZQ, SEP and VHS were previously described in detail [33,43,46,47]. In Figure 4, the two most intense bands of the PZQ appear at 1665–1621 cm^−1^, which correspond to the ν(C=O) stretching vibration mode. The spectra of sepiolite and montmorillonite showed intense bands at 1200–800 cm^−1^, corresponding to the ν(Si–O) and δ(MOHM’) (M, M’ = Al, Mg) vibrations [47].

In the interaction products prepared with sepiolite (Figure 4a) and montmorillonite (Figure 4b), the bands of the pristine PZQ were found, such as the bands that appeared at 1500–1400 cm^−1^, which are characteristic of the δ(CH) bands of bending vibrations. This corroborated that the drug is present in the drug–clay interaction products regardless of the solvent used in the elaboration process, but the bands of the PZQ are masked by the clay in all cases, since the clay is in a much higher proportion.

### 3.4. In Vitro Release Studies

**Figure 5:**
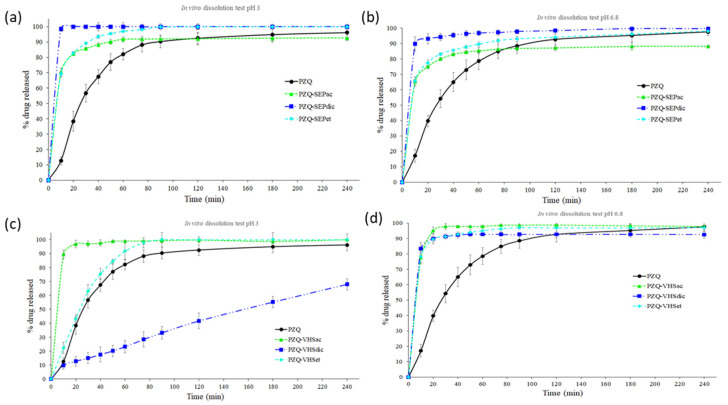
In vitro drug release profiles of sepiolite interaction products in HCl 0.001 M (**a**) and in simulated intestinal fluid (**b**), and of montmorillonite interaction products in HCl 0.001 M (**c**) and in simulated intestinal fluid (**d**); (mean values ± 6 SD; *n* = 7).

Drug release profiles of the interaction products with sepiolite (PZQ–SEPet, PZQ–SEPac and PZQ–SEPdic) and montmorillonite (PZQ–VHSet, PZQ–VHSac and PZQ–VHSdic) compared with the pristine PZQ were studied in sink conditions in acid aqueous medium with HCl 0.001 M (pH = 3) and in simulated intestinal fluid (pH = 6.8) (Figure 5).

The PZQ–SEP interaction products in acid medium revealed that all the products increased the dissolution rate of the PZQ drug. This enhance in the drug release profile is higher in the following order PZQ–SEPdic > PZQ–SEPet > PZQ–SEPac (Figure 5a). The same results were obtained in the SIF medium (Figure 5b). Therefore, the interaction products with sepiolite increase the dissolution rate of the PZQ greatly using any of the three solvents in the preparation method, with PZQ–SEPdic being the one that showed the best results in both media. This higher dissolution rate is due to the loss of crystallinity of PZQ that occurred during the intercalation in sepiolite. The amorphization overcomes the high cohesion energy in the packing of the PZQ crystal lattice [43] needed for its dissolution. In particular, this amorphization is higher in PZQ–SEPdic (Figure 2a) and, hence, its dissolution rate is the highest one.

The PZQ–VHS interaction products showed differences between them in acid medium (Figure 5c). In this medium, a strong increase in the dissolution rate of the drug was observed in the PZQ–VHSac product, whereas PZQ–VHSet showed only a slightly higher dissolution rate than the pristine PZQ. On the contrary, a surprising result of PZQ–VHSdic was found in acidic medium. In this case, the drug is released at a constant rate. This, in principle, leads to better control of plasma concentration and offers several advantages. Therefore, the PZQ–VHSdic in acidic medium showed a release that is interesting for new PZQ controlled release systems (Figure 5c). In simulated intestinal fluid, all the PZQ–VHS interaction products showed an increase in the dissolution rate with respect to the pristine PZQ drug. The material prepared with acetonitrile (PZQ–VHSac) presented the fastest release profile, followed by PZQ–VHSet and PZQ–VHSdic, which also presented a similar profile. In general, the increase in the dissolution rate of the PZQ–VHS interaction products also owes to the amorphization of PZQ. The peculiar behavior of PZQ–VHSdic at low pH can be due to a possible effect of the pH on the opening of the pores and interlayers of VHS that does not occur at higher pH, where the swelling is faster. Among the solvents used in this work, the dichloromethane is the only solvent non-miscible with water. It is likely that this behavior changed the macroscopic structure of the solid.

Moreover, in general, the PZQ–clay dissolution rate was higher in acidic medium (pH = 3) than in simulated intestinal fluid (pH = 6.8), in concordance with the results in no-sink conditions previously obtained in PZQ–clay interaction products prepared with ethanol [33]. Therefore, the interaction of the drug with the clay minerals induced an increase in dissolution rates with the independence of pH. This improvement was demonstrated in all interaction products with sepiolite in both media, and with montmorillonite in SIF medium. The interaction products with montmorillonite in acid medium showed a different behavior between them, with the PZQ–VHSac being the one with the highest dissolution rate. 

Therefore, according to the results obtained in vitro, PZQ–VHS and PZQ–SEP interaction products might improve the oral bioavailability of the drug by increasing both dissolution rate and amount of drug dissolved in the medium that simulates the stomach, obtaining new accelerated oral release systems of the PZQ. Moreover, the PZQ–VHSdic in the acidic medium could be a new controlled release system of the drug.

Subsequently, the experimental dissolution data of PZQ, PZQ–SEP and PZQ–VHS interaction products were fitted to various kinetic models in order to analyze the drug release. The correlation coefficient (R^2^) and Akaike Information Criterion (AIC) obtained from the dissolution model’s fitting are summarized in Tables S1 and S2. In general, the results showed that zero-order and first-order models were not appropriate to study these dissolution kinetics. In the same way, Square Root (Higuchi) and Power Law (Peppas) are not appropriate for the adjustments of the experimental dissolution values since there are no sufficient values lower than 63.2% of the drug release to adjust a kinetic [37]. In Tables S1 and S2, correlation coefficient and AIC values of PZQ, PZQ–SEPac, PZQ–SEPdic, PZQ–SEPet, PZQ–VHSac, PZQ–VHSet in acid and SIF media suggested that the Weibull model could be considered as an adequate model to describe the release kinetic, because the R^2^ obtained was the highest value and AIC value was the lowest once compared with the rest of the proposed models. The Weibull model presented an initial burst release [48,49], which is increased in the interaction products with clays. However, PZQ–VHSdic in an acid medium (pH = 3) showed a different dissolution profile, as can be seen in Table S2 and Figure 5c. In this case, the results suggested that the Cube Root (Hixson–Crowell) is the more accurate model to describe the release kinetics. Therefore, the PZQ–VHSdic IP in an acid medium (pH = 3) presented a release that is controlled by the dissolution rate of the drug particles and it is assumed that the particles of the interaction product are isometric and monodisperse. This geometric shape of the particles remains constant and there is a decrease in the surface area associated with the dissolution of the pharmaceutical form [50,51].

### 3.5. Solubility Studies 

The PZQ–SEPet and PZQ–VHSet showed an increase in the solubility compared to the pristine drug in the studied media. The results in the acid aqueous medium with HCl 0.001 M (pH = 3) indicated that the solubility of the PZQ–SEPet and PZQ–VHSet interaction products were similar in both cases (0.71 and 0.74 mg/mL, respectively). Similar results are obtained in simulated intestinal fluid (pH = 6.8), where the solubility of PZQ–SEPet and PZQ–VHSet was 0.61 and 0.65 mg/mL, respectively. Therefore, the interaction products enhanced the solubility of the drug, and this increase was in the range of 36–48% with respect to pristine PZQ (Table 1).

### 3.6. Cytotoxicity Studies

In vitro cytotoxicity tests in the HCT116 cell line were performed in PZQ, SEP, VHS, PZQ–SEPet and PZQ–VHSet products. The interaction products with ethanol were selected as a reference from the rest because they present an increase in the in vitro dissolution profile in sink conditions and in non-sink conditions, as demonstrated by previous work [33].

**Figure 6:**
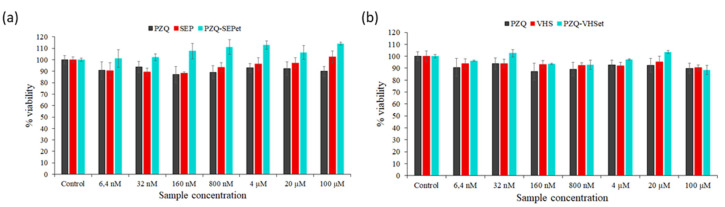
Cell viability of SEP and PZQ–SEPet (**a**), and VHS and PZQ–VHSet (**b**) compared to PZQ after 48 h of treatment. (Control: untreated cells in complete medium; mean values ± 8 SE; *n* = 3).

The raw material (PZQ, SEP and VHS) in concentrations between 6.4 nM and 100 μM were tested, demonstrating that they can be considered biocompatible toward HCT116 cell lines due to the fact that the percentage of the cell viability was slightly lower than the untreated cells in all cases (control) (Figure 6). 

In the PZQ–SEPet interaction products, the loss of cell viability was not observed in any of the concentrations tested, even a positive effect in cell growth was observed with respect to the control, and pure SEP and PZQ. Therefore, the PZQ–SEPet revealed its biocompatibility with cell and had a positive effect on the cell viability compared to raw PZQ and SEP solids in these ranges of concentrations (Figure 6a). PZQ–SEPdic and PZQ–SEPac products are expected to behave similarly.

PZQ–VHSet showed a slight loss of cell viability like pure products, up to a maximum of 11% in the highest concentration tested. In this case, a positive effect on cell growth was not observed, but the viability of the PZQ–VHSet interaction product was similar to that of the pristine drug and VHS, therefore it can be considered biocompatible according to the in vitro results (Figure 6b). Likewise, PZQ–VHSdic and PZQ–VHSac products are expected to have similar behavior.

### 3.7. Cell Cycle Studies

**Figure 7:**
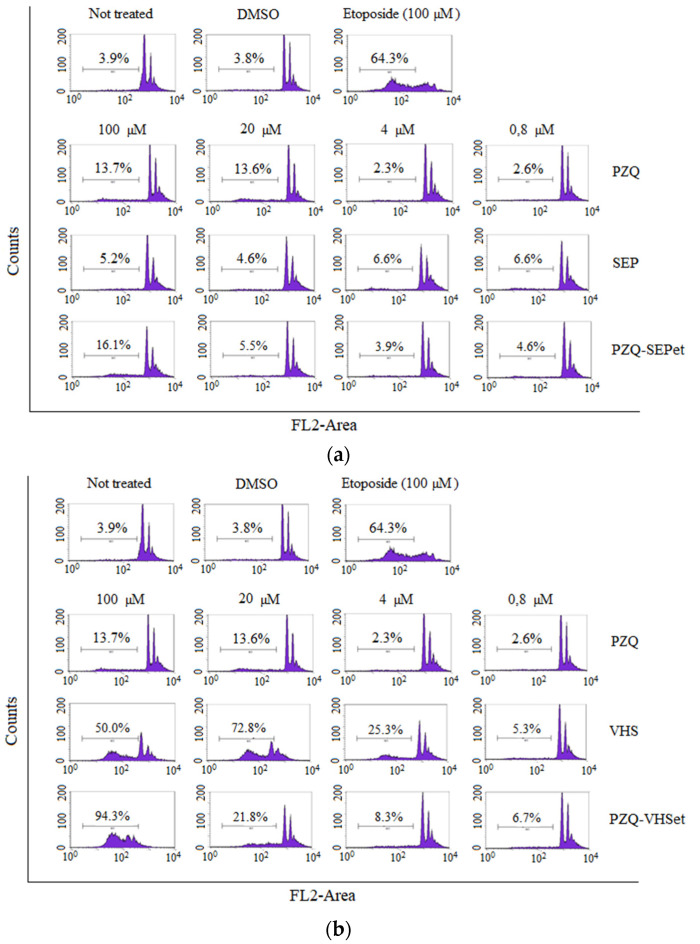
Cell cycle of the HCT116 line treated with the PZQ, SEP and PZQ–SEPet (**a**), and with the PZQ, VHS and PZQ–VHSet (**b**). The percentages indicate the number of cells in Sub-G1 (apoptotic or necrotic). Negative controls (Not treated and DMSO) and positive control (Etoposide) are also included.

The cell cycle tests by means of propidium iodide were performed to investigate the cell cycle corresponding to the Sub-G1 phase of the cells in contact with the interaction products prepared (PZQ–SEPet and PZQ–VHSet) and their raw materials (PZQ, SEP and VHS) (Figure 7). These studies allowed us to deduce if there is any alteration of any phase of the cell cycle despite the cell proliferation not being affected, after observing above that there is no significant loss in cell viability of the cells treated with the samples studied.

The control tests are shown in the upper part of the figure (Figure 7). Control tests demonstrated that, in comparison with cellular cycles of healthy, untreated cells (“not treated” cells in Figure 7), DMSO does not affect the cells, unlike etoposide (antineoplasic drug). As can be seen by the cell cycle analysis, the etoposide control group is severely affected and induces death to 64.3% of the cell population.

In Figure 7a, the results showed that the cell cycle was not affected when the cells were treated with PZQ, SEP and PZQ–SEPet, and only at high concentrations, the cell death was increased slightly, although not in a notable way. Furthermore, in none of the concentrations tested was it observed that the PZQ–SEPet interaction product produced a significantly higher percentage of cell death than that with the pure PZQ. 

The results of PZQ, VHS and PZQ–VHSet samples are shown in Figure 7b. The PZQ–VHSet interaction product showed that the cellular cycle of the cells was not affected, and cell death was not observed at low sample concentration (0.8–4 µM). At high concentrations (20–100 µM), affectation of the cell cycle of the cells was observed as well as greater cell death. This damage in the cell cycle is very high at the 100 µM concentration. This is the same in pure VHS clay, therefore, this occurs in the interaction product due to the presence of the clay mineral.

In summary, the PZQ–SEPet interaction product showed that it does not affect, in any concentration, the cellular cycle of the cells, and its behavior is similar to the pure drug. However, the PZQ–VHSet interaction product showed that it affects, at high concentrations, the cellular cycle, so only the lower concentrations (<0.8–4 µM) do not affect the Sub-G1 phase of the cells (Figure 7).

## 4. Conclusions

The interaction products PZQ–SEP and PZQ–VHS prepared with ethanol, dichloromethane and acetonitrile allowed us to carry out an effective interaction of the low water-soluble drug with the clays. Therefore, this described methodology that uses organic solvents and clays can be used to prepare drug–clay complexes, with drugs that have low aqueous solubility. The characterization using different techniques of these interaction products showed that the drug is amorphized, reducing its crystallinity.

In vitro drug release profiles of the interaction products with sepiolite compared with the pristine PZQ in sink conditions in acid aqueous medium (pH = 3) and in simulated intestinal fluid (pH = 6.8) showed that in all products the dissolution rate of the drug improved, and specifically the PZQ–SEPdic was the one that most increased the rate dissolution. In the interaction products with montmorillonite, in acid medium (pH = 3), the PZQ–VHSac improved the dissolution rate of the drug, and the PZQ–VHSdic showed a controlled release of PZQ, which can be very interesting for another type of modified drug delivery system. In SIF medium (pH = 6.8), all the PZQ–VHS interaction products increased the dissolution rate of the drug.

Cytotoxicity and cell cycle studies were performed in the interaction products prepared with ethanol, and the interaction products with dichloromethane and acetonitrile are expected to have similar behavior. The PZQ–SEPet interaction product was biocompatible with the HTC116 line cells, and it did not produce a decrease in cell viability or alterations in the cell cycle. However, the PZQ–VHSet showed a slight loss of cell viability like pure products, but in the cell cycle study, the affectation of the cell cycle and damage of the cells were observed at the highest concentration tested, so low concentrations should be used.

Therefore, the methodology used with organic solvents is an interesting technological strategy for effective interaction between praziquantel and clays. In general, according to the results obtained, the PZQ–SEP interaction products increased more the dissolution rates of drug, without producing cytotoxicity or alterations in the cell cycle of HTC116 cells. These systems should be considered as promising pharmaceutical systems to improve the bioavailability of water low-soluble of praziquantel.

## Figures and Tables

**Table 1:** Solubility values (in mg/mL) of praziquantel (PZQ), PZQ–SEPet and PZQ–VHSet and increase solubility (in %) with respect to that of PZQ in acid and SIF media (solubility mean values ± 0.07 SD; *n* = 3).

	HCl 0.001 M Medium, pH = 3		SIF Medium, pH = 6.8	
	Solubility (mg/mL)	Increase (%)	Solubility (mg/mL)	Increase (%)
PZQ	0.50		0.45	
PZQ–SEPet	0.71	42	0.61	36
PZQ–VHSet	0.74	48	0.65	44

## Supplementary Materials

The following are available online at https://www.mdpi.com/1999-4923/12/10/914/s1, Table S1: Statistical parameters corresponding to the fittings of all the experimental release results of PZQ and PZQ–SEP interaction products to the equations of distinct models, Table S2: Statistical parameters corresponding to the fittings of all the experimental release results of PZQ and PZQ–VHS interaction products to the equations of distinct models.
