# Supplementary material for: Praziquantel–Clays as Accelerated Release Systems to Enhance the Low Solubility of the Drug
Source: Pharmaceutics. 2020 Sep 24;12(10):914. doi: 10.3390/pharmaceutics12100914 (PMC7598598; doi:10.3390/pharmaceutics12100914)
Supplement: Supplementary file 1 [file pharmaceutics-12-00914-s001.pdf]

pharmaceutics

Supplementary Materials: Praziquantel–Clays as Accelerated Release Systems to Enhance the Low Solubility of the Drug

Ana Borrego-Sánchez, Rita Sánchez-Espejo, Fátima García-Villén, César Viseras, C. Ignacio Sainz-Díaz

Table S1. Statistical parameters corresponding to the fittings of all the experimental release results of PZQ and PZQ–SEP interaction products to the equations of distinct models.

Proposed models	Medium	Product	R^2	AIC
Zero Order	Acidic medium pH = 3	PZQ	0.5451	92.651
		PZQ–SEPac	0.3602	64.636
		PZQ–SEPdic	0.1153	8.527
		PZQ–SEPet	0.7613	43.354
	SIF medium pH = 6.8	PZQ	0.6017	88.472
		PZQ–SEPac	0.4405	63.787
		PZQ–SEPdic	0.6719	38.991
		PZQ–SEPet	0.6054	66.174
First Order	Acidic medium pH = 3	PZQ	0.8403	7.701
		PZQ–SEPac	0.4803	2.329
		PZQ–SEPdic	-	110.453
		PZQ–SEPet	0.9236	4.773
	SIF medium pH = 6.8	PZQ	0.9369	−0.923
		PZQ–SEPac	0.5581	−5.406
		PZQ–SEPdic	0.9385	−1.962
		PZQ–SEPet	0.9252	−6.168
Cube Root (Hixson Crowell)	Acidic medium pH = 3	PZQ	0.7442	11.352
		PZQ–SEPac	0.4403	−1.466
		PZQ–SEPdic	-	108.167
		PZQ–SEPet	0.8729	−13.688
	SIF medium pH = 6.8	PZQ	0.8364	6.634
		PZQ–SEPac	0.5183	−6.689
		PZQ–SEPdic	0.8920	−14.695
		PZQ–SEPet	0.8341	−5.056
Square Root (Higuchi) (Release ≤ 63.2%[a] / all data[b])	Acidic medium pH = 3	PZQ	−/0.7096	−/86.052
		PZQ–SEPac	−/0.5382	−/61.050
		PZQ–SEPdic	−/0.2128	−/7.240
		PZQ–SEPet	−/0.6006	−/67.055
	SIF medium pH = 6.8	PZQ	−/0.7808	−/81.902
		PZQ–SEPac	−/0.6237	−/59.423
		PZQ–SEPdic	−/0.8343	−/31.477
		PZQ–SEPet	−/0.7761	−/59.941
Power Law (Peppas) (Release ≤ 63.2%[a] / all data[b])	Acidic medium pH = 3	PZQ	−/0.7498	−/19.334
		PZQ–SEPac	−/0.7352	−/−60.224
		PZQ–SEPdic	−/0.3832	−/−114.984
		PZQ–SEPet	−/0.7868	−/−55.920
	SIF medium pH = 6.8	PZQ	−/0.8220	−/−24.407
		PZQ–SEPac	−/0.8059	−/−61.710
		PZQ–SEPdic	−/0.9611	−/−102.962

		PZQ–SEPet	–/0.9042	–/–64.512
Weibull	Acidic medium pH = 3	PZQ	0.8999	−17.443
		PZQ–SEPac	0.8136	−41.182
		PZQ–SEPdic	-	-
		PZQ–SEPet	0.9522	−26.455
	SIF medium pH = 6.8	PZQ	0.9617	−29.813
		PZQ–SEPac	0.8665	−46.846
		PZQ–SEPdic	0.9544	−53.204
		PZQ–SEPet	0.9925	−67.154

[a] Values obtained considering only drug released ≤ 63.2%; [b] Values obtained considering the complete drug released until 100%.

Table S2. Statistical parameters corresponding to the fittings of all the experimental release results of PZQ and PZQ–VHS interaction products to the equations of distinct models.

Proposed models	Medium	Product	R^2	AIC
Zero Order	Acidic medium pH = 3	PZQ	0.5451	92.651
		PZQ–VHSac	0.3102	42.590
		PZQ–VHSdic	0.9945	34.496
		PZQ–VHSet	0.9433	53.656
	SIF medium pH = 6.8	PZQ	0.6017	88.472
		PZQ–VHSac	0.1516	65.619
		PZQ–VHSdic	0.2148	47.386
		PZQ–VHSet	0.3864	59.811
First Order	Acidic medium pH = 3	PZQ	0.8403	7.701
		PZQ–VHSac	0.4435	18.256
		PZQ–VHSdic	0.9933	11.796
		PZQ–VHSet	0.9637	-5.734
	SIF medium pH = 6.8	PZQ	0.9369	-0.923
		PZQ–VHSac	0.1973	20.774
		PZQ–VHSdic	0.2465	-6.151
		PZQ–VHSet	0.5896	7.904
Cube Root (Hixson Crowell)	Acidic medium pH = 3	PZQ	0.7442	11.352
		PZQ–VHSac	0.4228	2.529
		PZQ–VHSdic	0.9981	-60.378
		PZQ–VHSet	0.9944	-8.505
	SIF medium pH = 6.8	PZQ	0.8364	6.344
		PZQ–VHSac	0.1842	9.836
		PZQ–VHSdic	0.2359	−12.633
		PZQ–VHSet	0.5256	0.171
Square Root (Higuchi) (Release ≤ 63.2%[a] / all data[b])	Acidic medium pH = 3	PZQ	–/0.7096	–/86.052
		PZQ–VHSac	–/0.4555	–/44.734
		PZQ–VHSdic	–/0.9749	–/51.094
		PZQ–VHSet	–/0.6599	–/87.625
	SIF medium pH = 6.8	PZQ	–/0.7808	–/81.903
		PZQ–VHSac	–/0.2749	–/63.892
		PZQ–VHSdic	–/0.3630	–/45.085
		PZQ–VHSet	–/0.9750	–/–39.245
Power Law (Peppas) (Release ≤ 63.2%[a] / all data[b])	Acidic medium pH = 3	PZQ	–/0.7498	–/19.334
		PZQ–VHSac	–/0.6512	–/–78.567
		PZQ–VHSdic	–/0.9741	–/–41.975

pharmaceutics

		PZQ–VHSet	–/0.7786	–/–23.972
	SIF medium pH = 6.8	PZQ	–/0.8220	–/–24.407
		PZQ–VHSac	–/0.4655	–/–56.212
		PZQ–VHSdic	–/0.5760	–/–76.253
		PZQ–VHSet	–/0.7534	–/–66.999
Weibull	Acidic medium pH = 3	PZQ	0.8999	−17.443
		PZQ–VHSac	0.7762	−34.075
		PZQ–VHSdic	0.9549	−31.442
		PZQ–VHSet	0.9948	−35.075
	SIF medium pH = 6.8	PZQ	0.9617	−29.813
		PZQ–VHSac	0.5524	−25.790
		PZQ–VHSdic	0.6120	−49.126
		PZQ–VHSet	0.8789	−44.003

[a] Values obtained considering only drug released ≤ 63.2%; [b] Values obtained considering the complete drug released until 100%.
